# Involvement of the PINK1/PARKIN pathway in enhancing mitochondrial function and mitophagy in reserpine-induced fibromyalgia mice through strength exercise and coenzyme Q10

**DOI:** 10.1007/s00421-025-05990-0

**Published:** 2025-12-02

**Authors:** Hoda M. Moghazy, A. A. Seham, Motamed Mahmoud, Sahar M. Gebril, Dina M. Monir

**Affiliations:** 1 Medical Physiology Department, Faculty of Medicine, Sohag University, University Street, Sohag, 82515 Sohag Egypt; 2 Physiology Department, Faculty of Medicine, Sohag University, Sohag, Egypt; 3 Animal Behavior and Husbandry Department, Faculty of Veterinary Medicine, Sohag University, Sohag, Egypt; 4 Histology and Cell Biology Department, Faculty of Medicine, Sohag University, Sohag, Egypt

**Keywords:** Fibromyalgia, Climbing exercise, Coenzyme Q10, Mitochondria, Mitophagy, PINK1, PARKIN

## Abstract

**Purpose:**

To assess the impact of reserpine-induced fibromyalgia and evaluate the potential effects of resistance exercise or CoQ10 administration on muscle strength, structure, and expression of mitochondrial markers in adult mice. Central to this investigation is an exploration of the molecular mechanisms underlying mitophagy via the PINK1/Parkin pathway.

**Methods:**

This study sought to elucidate the Impact of 4 weeks of either climbing exercise or coenzyme Q10 (CoQ10) supplementation (10 mg/kg, administered once daily) on skeletal muscle and mitochondrial functions within a reserpine-induced fibromyalgia (FM) mouse model. Evaluation encompassed the assessment of key mitochondrial markers, including PTEN-induced kinase 1 (PINK1), PARKIN, Mitofusion2, cytochrome c oxidase, citrate synthase, and fibronectin type III domain-containing protein 5 (FNDC5), alongside morphological examinations of the gastrocnemius muscle.

**Results:**

Climbing exercise significantly improved fibromyalgia (FM)-like symptoms and enhanced the expression of mitochondrial marker genes in the gastrocnemius muscle. Histological and ultra-structural studies showed nearly normal muscle fiber structure, banding patterns, mitochondria shape and size, and a notable reduction in collagen fibrosis compared to FM. CoQ10 supplementation also improved mitochondrial gene expression but did not significantly affect FNDC gene expression. Ultrastructural analysis revealed mostly normal muscle fibers with regular banding, though some areas showed disturbances with multiple sub-sarcolemmal and interfibrillar mitochondria.

**Conclusion:**

This study underscores the efficacy of both resistance exercise and CoQ10 supplementation as viable strategies for improving FM-related symptoms and enhancing mitochondrial function in mice.

**Supplementary Information:**

The online version contains supplementary material available at 10.1007/s00421-025-05990-0.

## Introduction

Fibromyalgia (FM) ranks as the third most prevalent musculoskeletal condition, following lumbar pain and osteoarthritis, with its prevalence correlating with age, peaking notably between 50 and 60 years (Laroche et al. 2019). Characterized by debilitating muscle pain significantly compromising the quality of life, FM manifests as diffuse chronic musculoskeletal pain accompanied by myopathy, fatigue, sleep disturbances, cognitive dysfunction, anxiety, and depression (Siracusa et al. 2021). Notably, muscle strength diminishes progressively throughout the disease. The precise etiology of FM remains unknown, with factors, such as genetic predisposition, environmental influences, hormonal imbalances, and neural alterations contributing to its onset (Siracusa et al. 2021). Despite this ongoing inquiry, mitochondrial dysfunction and coenzyme Q10 (CoQ10) deficiency emerge as common denominators in its pathogenesis. Aberrant mitochondria, featuring anomalies in oxidative phosphorylation mitochondrial complexes, diminished ATP levels, and CoQ10 deficits, have been discerned in muscle and skin biopsies from FM patients. Moreover, oxidative stress and mitochondrial dysfunction may foster peripheral and central sensitization, thereby elucidating the widespread chronic pain experienced by FM patients (Assavarittirong et al. 2022).

Maintaining mitochondrial homeostasis assumes great importance in preserving muscle function despite metabolic and environmental stressors. Mitochondrial homeostasis depends on mitochondrial dynamics and mitophagy mechanisms (Wan et al. 2022). Mitochondrial dynamics, governed by mitochondrial fission and fusion proteins, involve integral players such as Mitofusins 1 and 2 (Mfn 1, Mfn 2), orchestrating outer mitochondrial membrane fusion to facilitate mitochondrial continuity (Sygitowicz and Sitkiewicz 2022). Mitophagy, a specialized autophagic pathway, is triggered under stress conditions. Upon encountering a loss in membrane potential, mitochondrial PTEN-induced putative kinase 1 (PINK1) stabilizes on the outer mitochondrial membrane, recruiting Parkin to induce ubiquitin localization on outer mitochondrial membrane targets. Subsequently, adapter protein sequestosome1 (p62) engages with mitochondrial ubiquitin and microtubule-associated protein light chain 3 (LC3) on the autophagic phagophore membrane, thereby facilitating the encapsulation and degradation of dysfunctional mitochondria within autophagosomes (Chen 2018; Zheng et al. 2024).

Exercise stands as a cornerstone intervention, eliciting numerous benefits, including enhanced maximum oxygen consumption, increased skeletal muscle mass, augmented muscle protein synthesis, and improved muscle function (Joanisse et al. 2020). Autophagy emerges as a putative mechanism underlying exercise-induced muscle plasticity, while myokine irisin demonstrates close associations with mitochondrial genes and proteins regulating mitochondrial function (Sanchez et al. 2014; He et al. 2020). Cytochrome c oxidase, a pivotal enzyme in the mitochondrial electron transport chain, couples electron transport to oxygen, generating a proton gradient crucial for ATP synthesis (Čunátová et al. 2020). Meanwhile, citrate synthase (CS) governs an essential step in the Krebs cycle, catalyzing the condensation of acetyl CoA with oxaloacetate into citrate. CS activity predicts maximal aerobic capacity, linking cellular and systemic metabolism (Mankowski et al. 2023; Angonese et al. 2022). Coenzyme Q10 (CoQ10), functioning as an electron carrier within the mitochondrial respiratory chain, facilitates oxidative phosphorylation. Additionally, its antioxidant properties in the fully reduced ubiquinol form, alongside reported anti-inflammatory effects and involvement in DNA replication and repair, underscore its multifaceted role (Porter et al. 2015).

Despite these insights, myopathy and muscle strength studies in FM remain limited. This study attempts to assess the impact of reserpine-induced fibromyalgia and evaluate the potential effects of resistance exercise or CoQ10 administration on muscle strength, structure, and expression of mitochondrial markers in adult mice. Central to this investigation is an exploration of the molecular mechanisms underlying mitophagy via the PINK1/Parkin pathway.

## Materials and methods

### Animals and experimental setup

Fifty male C57Black/6 (C57BL/6) mice, aged 12 ± 4 weeks and weighing 30 ± 10 g, were housed in groups of five within polycarbonate cages (20 × 32 × 20 cm) in the animal research laboratory at the Faculty of Medicine, Sohag University. The housing conditions maintained a standard light–dark cycle, with a temperature set at 23 ± 2 °C and humidity at 60 ± 5%. Mice were given ad libitum access to standard chow and tap water throughout the study. Experimental procedures were conducted consistently at the same time each day to ensure uniform handling and environmental conditions. The mice underwent a 1-week acclimatization period to the laboratory environment before the start of experiments, during which they were habituated to the light/dark cycle. Sample size was calculated using program G Power (Faul et al. 2007) based on Cohen’s principles (Charan and Kantharia 2013). Subsequently, mice were randomly allocated to one of five experimental groups (*n* = 10 per group), as detailed in Table 1 and the schematic provided below.

**Table 1:** Experimental groups

Group	Treatment
Control	Injected with saline solution (0.1 ml/mouse, subcutaneously) once daily from day 1 (D1) to day 3 (D3) (Control group)
Exercise control	A climbing exercise regimen was initiated from D4 through D32 (EX/control group)
Fibromyalgia (reserpine-treated)	Administered reserpine injection (5 mg/kg) at a volume of 0.1 ml subcutaneously once daily from D1 to D3 (FM group)
Fibromyalgia/exercise (reserpine-exercise)	Administration of reserpine injection followed by a climbing exercise regimen starting on D4 till D32 (FM/EX)
Fibromyalgia/CoQ10 (reserpine-CoQ10)	Reserpine injection was followed by daily oral administration of CoQ10 (10 mg/kg) from D4 to D32 (FM/CoQ10)

*FM* fibromyalgia, *CoQ10* coenzyme Q10

### Climbing exercise protocol

The mice climbed the vertical ladder for 5 weeks (1-week adaptation, 4 weeks training). The exercise was accomplished by utilizing a 1-m ladder with a 1.5-cm grid and inclined at 85°. Initially, the mice climbed the ladder without weights for a week to become adapted. For the first week of training, a weight equivalent to 10% of their body weight was attached to the base of their tail, and the resistance was progressively increased to 30% in the 2nd week, 40% in the 3rd week, and 50% in the 4th week. When the mice reached the top of the ladder, they were allowed to rest for 90 s. The training session was stopped when the mice succeeded in climbing the ladder for eight repetitions (Kim 2019).

Behavioral assessments were conducted on day 33 after the exercise regimen, and CoQ10 treatment ended. The following standardized behavioral tests were employed to evaluate fibromyalgia-like symptoms and muscle strength:

**Hot-plate test:** Pain threshold, indicative of fibromyalgia state, was assessed using the Hot-Plate Test (HPT). The hot-plate surface temperature was set to 50 ± 0.5 °C before testing, with a maximum test duration of 60 s to prevent tissue damage. Latency time, defined as the interval between placing the animal on the hot-plate surface and the occurrence of paw licking or jumping to avoid thermal pain, was measured. Additionally, the number of jumps was recorded upon placement of each mouse on the hot-plate apparatus (Reddy et al. 2021).

**Forced swim test:** Depressive-like behavior in mice with experimental Fibromyalgia (FM) was evaluated using the Forced Swim Test (FST), following a previously established protocol (Wulf et al. 2022). Mice were subjected to a 6-min trial period in a cylindrical container filled with water maintained at 30 °C. Immobility, characterized by the absence of all movements except those required to keep the head above water, was quantified in seconds during each 1-min interval.

**Open-field test:** Locomotion, anxiety, and stereotypical behaviors, including grooming and rearing, were assessed using the Open-Field Test (OFT). The test arena, typically a 42 × 42 × 42 cm polyvinyl chloride (PVC) box, was divided into defined sections. Movement within and around the central and peripheral areas of the box was monitored using a camera over a 5-min exploration period. The duration spent in the central area was recorded within this period (Kraeuter et al. 2019).

**Weights test:** Muscle strength was evaluated using weights ranging from 20 to 98 g. Mice grasped weights by their tail base and attempted to hold each weight for 3 s. The duration of successful weight holding was noted, with a 10-s rest period before another attempt. Failure in three consecutive attempts terminated the trial. The final score was calculated as the product of the heaviest weight held for 3 s and its duration. An intermediate value was assigned if the heaviest weight was dropped before 3 s (Deacon 2013).

**Kondziela’s inverted screen test:** Muscle strength in mice was assessed using all four limbs with Kondziela’s Inverted Screen Test. Mice were positioned at the center of a wire mesh screen, which was then gradually inverted over 2 s. The time was recorded until the mouse fell off or reached the 60-s criterion. Scores ranged from 1 to 4 based on falling times (Vogel et al. 2021).

### Sampling procedure

Upon completion of the study, all animals were anesthetized using 2% isoflurane inhalation anesthesia. A surgical incision was performed in the chest wall to expose the heart, facilitating the perfusion of 15–20 ml of PBS at 37 °C through the left ventricle with a snap cut in the right auricle. During perfusion of PBS, the left gastrocnemius muscle was rapidly dissected and trimmed on ice, rapidly frozen in liquid nitrogen, and then stored at − 80 °C for PCR. After PBS perfusion, 20 ml of neutral buffered solution of 10% formalin was perfused, followed by dissection and trimming of the right gastrocnemius muscle and divided into pieces that were further preserved in neutral buffered solution of 10% formalin for 48 h for light microscope and other pieces preserved in solution containing 2% PFA and 2.5% glutaraldehyde for electron microscope studies. This was done for mice in all experimental groups.

### Reverse transcription and quantitative PCR

Reverse transcription and quantitative real-time polymerase chain reaction (qPCR) were conducted using the GeneJET RNA Purification Kit for RNA extraction and Maxima SYBR Green qPCR Master Mix for reverse transcriptase real-time PCR reaction kits, both sourced from Thermo Scientific (Waltham, Massachusetts, USA). PCR samples were stored at − 80 °C in liquid nitrogen. Total muscle RNA extraction followed established protocols, with RNA concentration determined via nanodrop spectrophotometry (Thermo Fisher Scientific, Waltham, Massachusetts, USA). Subsequently, cDNA synthesis utilized RNA reverse transcriptase kits. The prepared cDNA underwent analysis with MAXIMA SYBR Green qPCR Master Mix under the following cycling conditions: 1 cycle at 95 °C for 10 min; 40 cycles of 95 °C for 15 s, 60 °C for 30 s, and 72 °C for 30 s; followed by one cycle at 95 °C for 15 s, 60 °C for 1 min, and 95 °C for 15 s. Gene expression analysis in skeletal muscle utilized specific primers targeting genes, such as PTEN-induced kinase 1 (PINK1), PARKIN, Mitofusion2, Cytochrome c oxidase, Citrate synthase, and Fibronectin type III domain-containing protein 5 (FNDC5) (Table 1). Fold expression was calculated relative to the housekeeping gene β-actin using the formula fold = 2^−∆∆*CT*^.

For light microscopy evaluation, 5 × 10 mm gastrocnemius muscle tissue pieces formalin-fixed were washed in running tap water for 1 h and then underwent automated processing utilizing a Leica TP1020 tissue processor (Leica Biosystems, Buffalo Grove, Illinois, USA). Paraffin-embedded tissue blocks were sectioned at 4 µm thickness using a rotary microtome and stained with hematoxylin and eosin (H&E) for histopathological Evaluation and Sirius red stain for collagen fiber demonstration (Al-Zahrani et al. 2023). Examination and image capturing of stained sections were performed using a Leica Microsystems, Germany’s Leica Microscope DFC310 FX 1.4-megapixel digital color camera with the Leica software application package, LAS EZ V 3.4.0.

For transmission electron microscopy, following fixation in 2% PFA and 2.5% glutaraldehyde, tissue samples underwent three washes in 0.1M PBS for 5 min before being post-fixed in 1% osmium tetroxide in 100 ml PBS for additional 2 h. Thirty minutes were spent to dehydrate in ethanol concentrations of 70%, 80%, 90%, and 100%. Once with 50% (v/v) and 100% epoxy resin (2 h each at room temperature), the surfaces were cleaned twice for 15 min with propylene oxide. At 60 °C for 72 h, the tissue was encapsulated in epoxy resin. Subsequently, fixed samples were sectioned into small pieces (approximately 2 × 2 mm) and processed to obtain semi-thin sections (250–500 nm thickness) using a microtome (RMC PT-X Leica Biosystems, Buffalo Grove, Illinois, USA). Ultra-thin sections (70 nm thickness) were then cut using a diamond knife and mounted on nickel grids for further analysis (Gebril et al. 2020; Nicola et al. 2024) (Table 2).

**Table 2:** List of forward and reverse primers for target and housekeeping genes

Gene	Sequence (5′–3′)	References	Accession number
PINK1	F—GCTTGCCAATCCCTTCTATG R—CTCTCGCTGGAGCAGTGAC	Zhou et al. (2019); Chang et al. (2022)	MP202248
Mitofusin-2	F—GAAGTAGGCAGTCTC CATCG R—AACATCGCTCAGCCTGAACC	Wang et al. (2019); Liu et al. (2021)	NM-133201.1
PARK	F—CCAGAGGAAAGTCACCTGCGAA R—CTTCTCCAAGGATCCTGAAGTGATG	Peker et al. (2022)	NM_016694
Cytochrome c oxidase	F—CAGAACCAGACGCGTAACTGCT R—GCTGATGGGACACAGTGAATGG	Quinn et al. (2022)	MR201462
Citrate synthetase	F—ATGCAGAGGGAATGAACCGAGC R—GAGTCAATGGCTCCGATACTGC	Xie et al. (2022)	NM_027945
FNDC5	F—GATGTCCTGGAGGATGAAGTGG R—GTGGTGGTGTTCACCTCCTGAA	Yan et al. (2022)	NM_027402
β-Actin	F—GGCTGTATTCCCCTCCATCG R—CCAGTTGGTAACAATGCCATGT	López-Muñoz et al. (2023)	NM_007393

Light and electron microscopy examination

### Statistical analysis

Data were analyzed using STATA version 17.0 (Stata Statistical Software: Release 17.0 College Station, TX: StataCorp LP.). Shapiro–Wilk normality test was used to determine the distribution of different variables. Quantitative data were represented by mean, standard deviation, median, and range. Data were analyzed using a one-way ANOVA test to compare the means of five groups with the post hoc Bonferroni test. When the data were not normally distributed, the Kruskal–Wallis test was used to compare the five groups, and the Mann–Whitney test was used to compare two groups. *p* value was considered significant if it was < 0.05.

## Results

### Reserpine-induced behavioral and pathophysiological changes

**Fig. 1:**
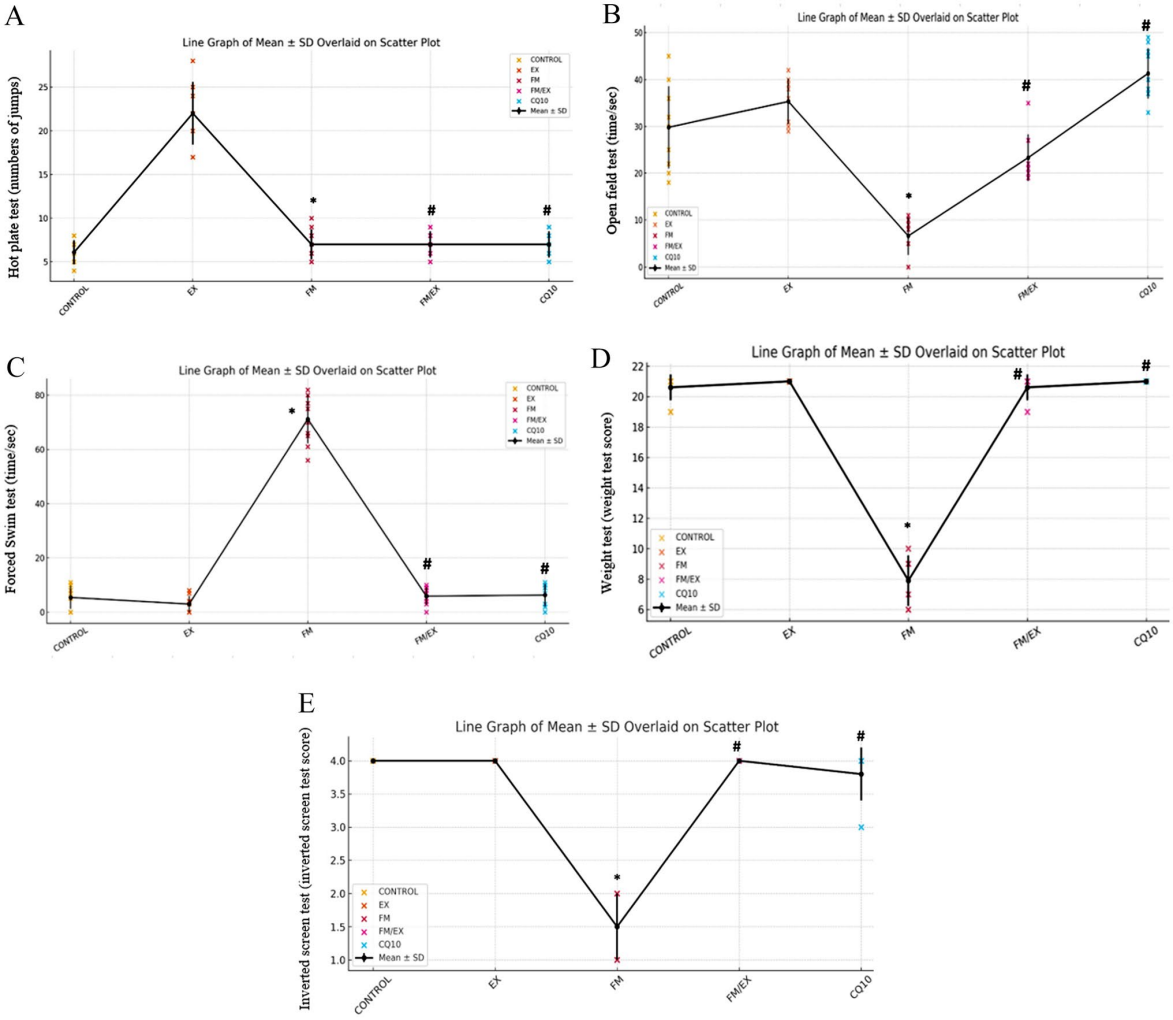
Effect of resistance exercise or coenzyme Q10 on reserpine-induced behavioral changes. **A** Boxplot graph showing distribution of hot-plate test by studied group: number of jumps observed in different groups. **B** Boxplot graph showing distribution of open-field test by studied group: time spent in the central area (seconds) in different groups. **C** Boxplot graph showing distribution of forced swimming test by studied groups: immobility time (seconds) in different groups. **D** Bar chart showing mean of weight test by studied group: score of the weights on D3 and at the end of the study among groups. **E** Bar chart showing mean of inverted screen test by studied group: score of the test study in all groups. Data are presented as means for reserpine-treated, resistance exercise, or coenzyme Q10 treatment (*n* = 10/group). *p < 0.05

The behavioral assessments provided fibromyalgia-related pathophysiological alterations after reserpine administration. Notably, there was a marked increase in pain sensitivity, exemplified by a substantial rise in the number of jumps (*p* < 0.0001) observed in the hot-plate test in reserpine-injected mice compared to the untreated control group (Fig. 1A). Additionally, reductions in locomotion and heightened anxiety-like behavior were evident, as indicated by a significantly decreased mean duration spent in the central arena during the open-field test (*p* < 0.0001) in reserpine-treated group compared to the control group (Fig. 1B). Furthermore, reserpine injection elicited observable depression-like behavior, evident from a significantly prolonged mean immobility time (*p* < 0.0001) in the forced swimming test in reserpine-treated group compared to the control group (Fig. 1C). Moreover, both the weights test and the inverted screen test scores demonstrated a significant decrease (*p* < 0.001) in a reserpine-treated group compared to the control group (Fig. 1D, E), indicative of reduced muscle strength following reserpine injection.

### Effect of reserpine, resistance exercise, or coenzyme Q10 on reserpine-induced behavioral and pathophysiological alterations

In this investigation, the climbing exercise regimen and administration of coenzyme Q10 (CoQ10) at a dose of 10 mg/kg for 4 weeks mitigated the physical manifestations induced by reserpine injection. In reserpine-injected mice, following either the climbing exercise program or CoQ10 administration for 4 weeks, pain sensitivity, locomotion, and anxiety and depression-like behaviors exhibited marked improvement. Specifically, the mean number of jumps in the hot-plate test and the mean immobility time in the forced swimming test significantly decreased (*p* < 0.0001). The mean time spent in the center of the open-field test significantly increased (*p* < 0.0001) compared to the reserpine-treated group. Importantly, these findings were nonsignificant (*p* > 0.05) when compared with those of the control group (Fig. 1A–C). Additionally, muscle strength was restored in reserpine-injected mice following 4 weeks of either the climbing exercise program or CoQ10 administration, with significant increases in the scores of both the weights test and the inverted screen test compared to the reserpine-treated group (*p* < 0.001). Conversely, these end-of-study scores were statistically nonsignificant (*p* > 0.05) compared to the control group (Fig. 1D, E). As anticipated, behavioral test results remained within the normal range and unchanged following the exercise program in healthy mice. There was a statistically insignificant difference in the results of the hot-plate test, open-field test, forced swimming test, weights test, and inverted screen test in the exercise-control group compared to the control group (*p* = 1.0, 0.41, 1.0, 0.45, 1.0, respectively) (Fig. 1D, E). To sum up, both the climbing exercise regimen and CoQ10 administration effectively mitigated the physical signs induced by reserpine injection, leading to notable improvements in pain sensitivity, locomotion, and anxiety and depression-like behaviors. These findings underscore the potential therapeutic benefits of these interventions in managing fibromyalgia-related pathophysiological changes.

### Effect of reserpine injection on mRNA expression of mitochondrial markers and FNDC genes in the gastrocnemius muscle

**Fig. 2:**
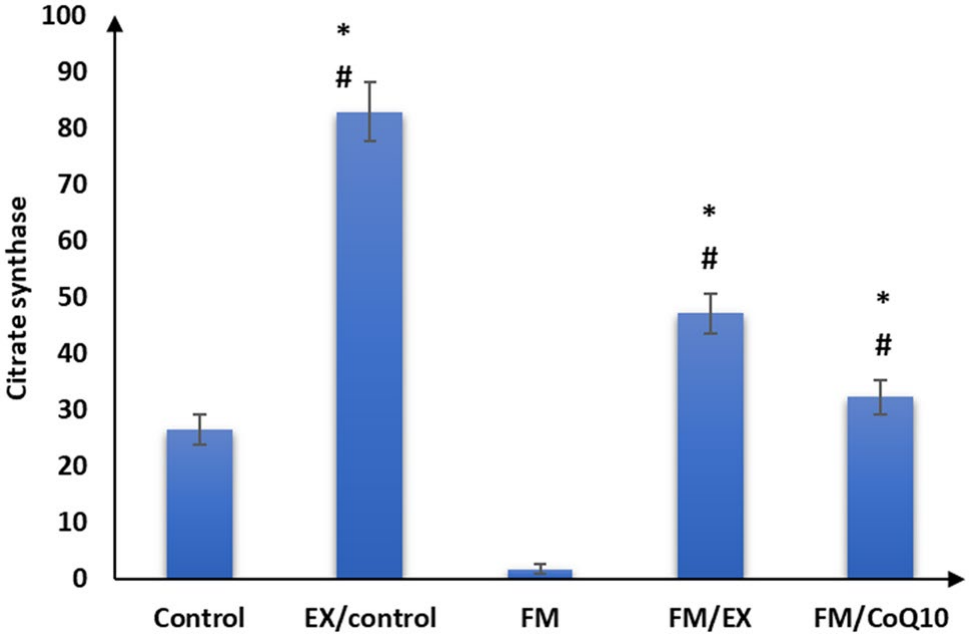
Relative expressions of cytochrome C oxidase compared to housekeeping gene in qPCR

**Fig. 3:**
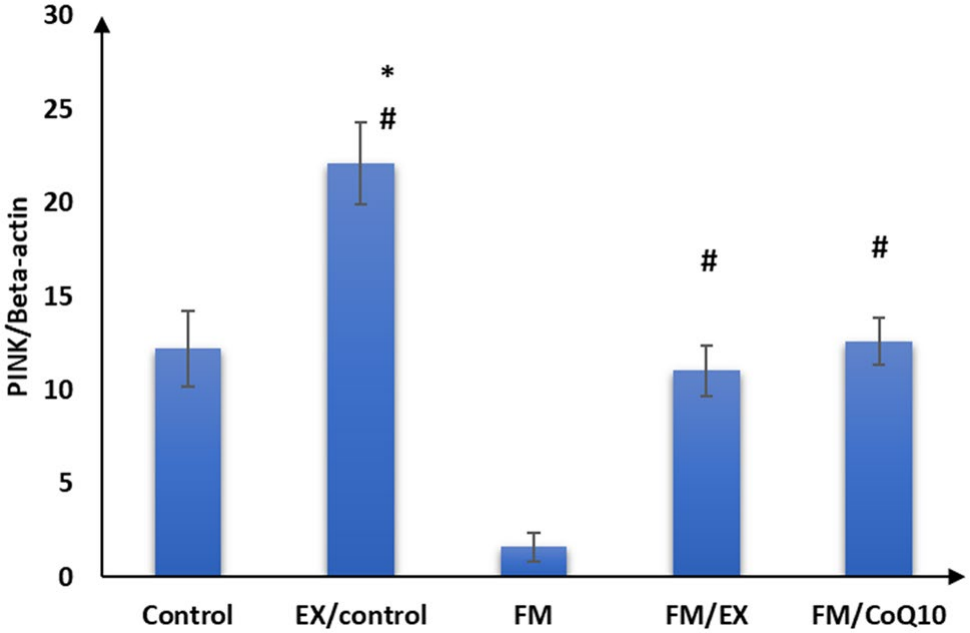
Citrate synthase compared to housekeeping genes in qPCR

**Fig. 4:**
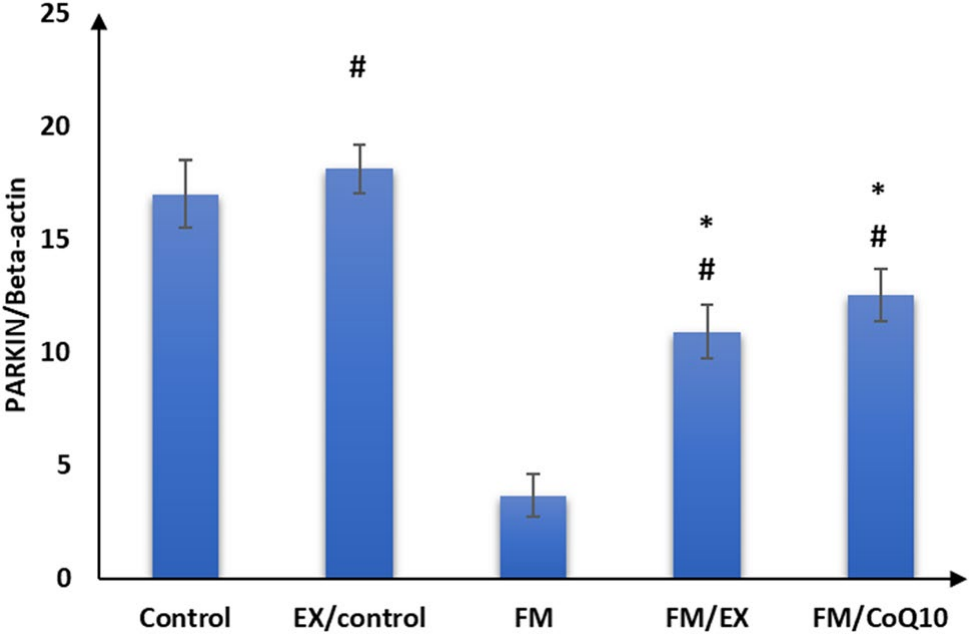
PINK compared to housekeeping gene in qPCR

**Fig. 5:**
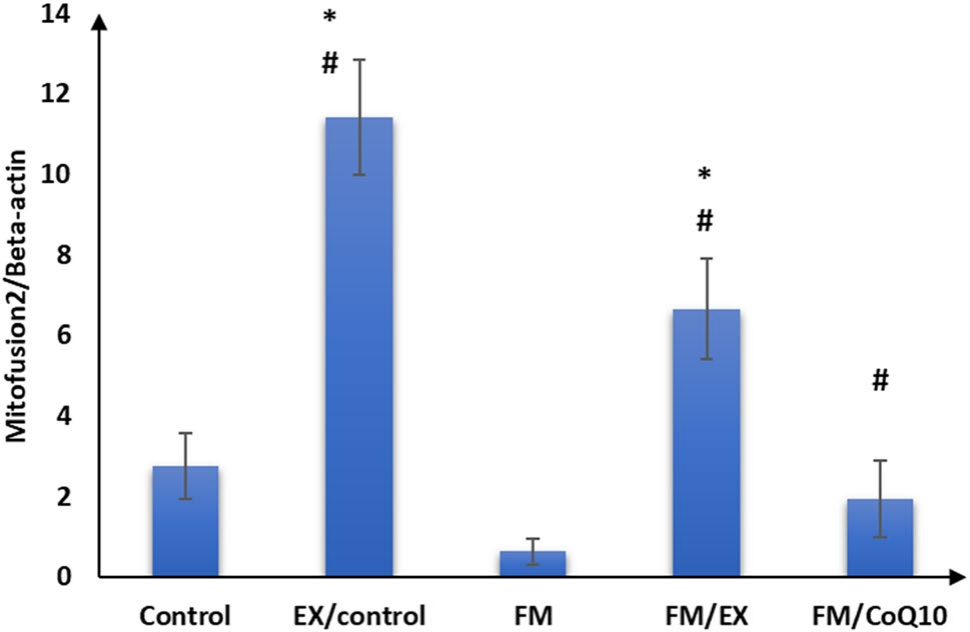
PARKIN compared to housekeeping gene in qPCR

**Fig. 6:**
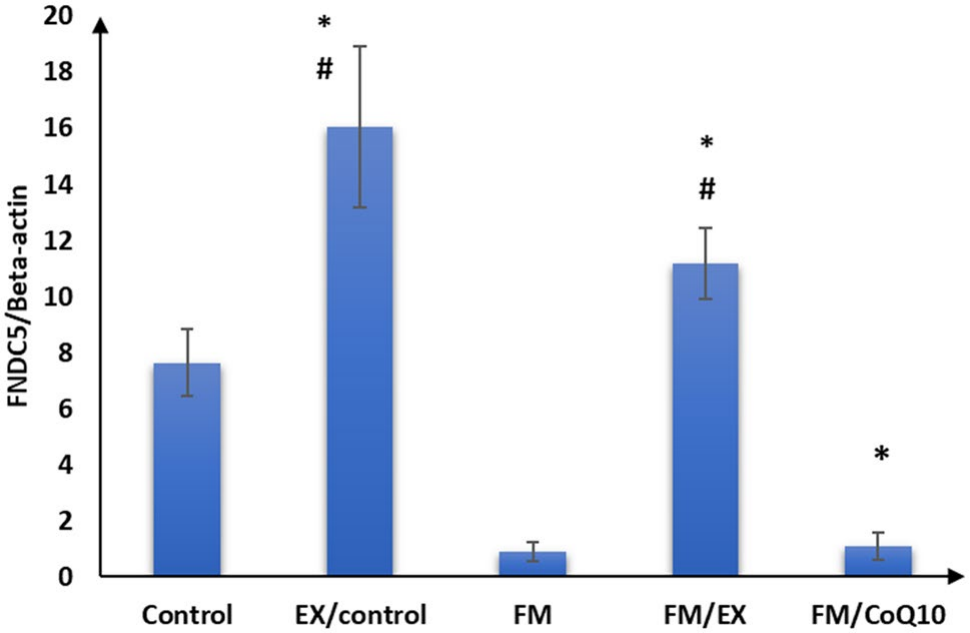
Mitofusion 2 compared to housekeeping gene in qPCR

**Fig. 7:**
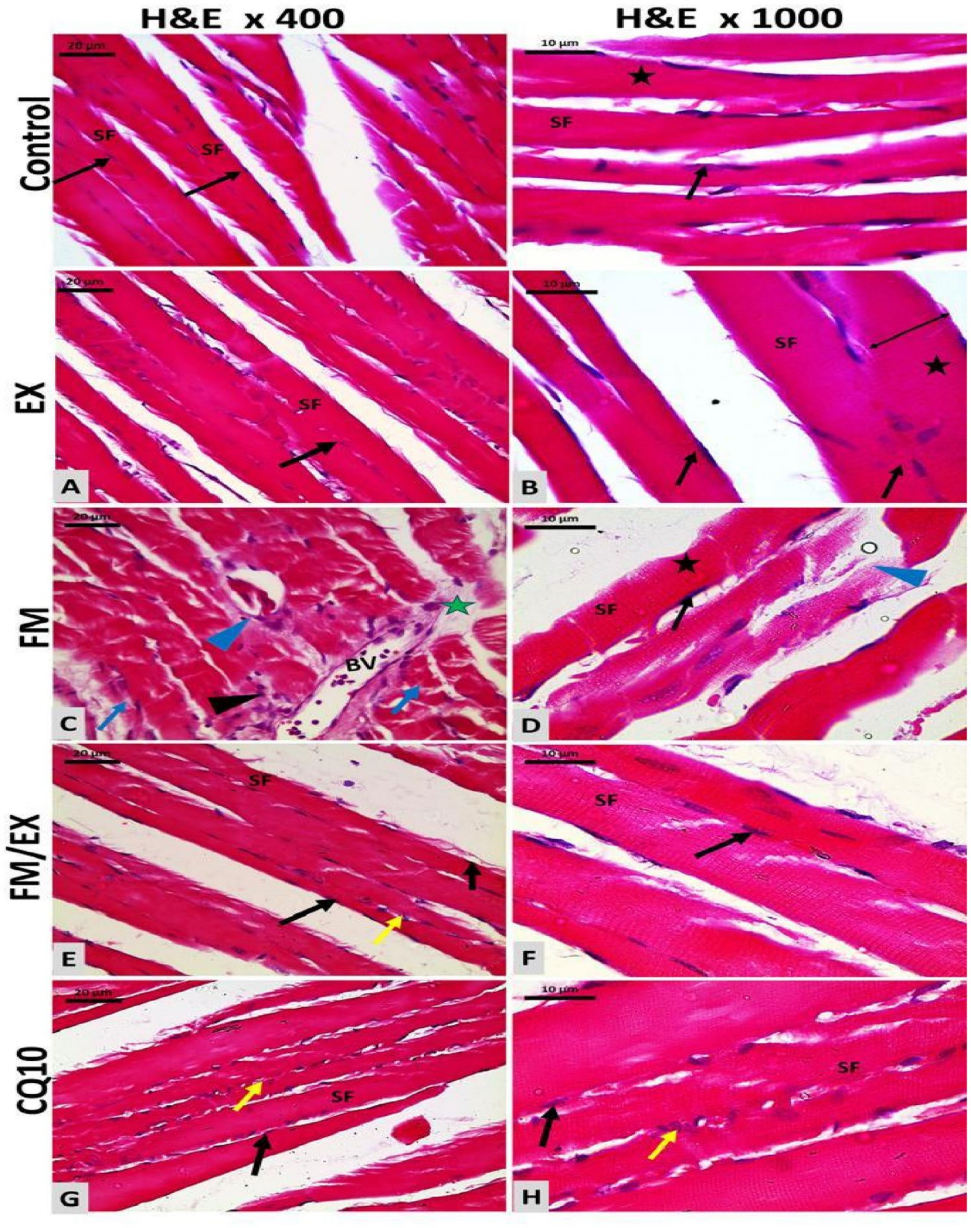
FNDC5 compared to housekeeping gene in qPCR

Subsequently, we evaluated the impact of reserpine injection on mitochondrial functional markers in the gastrocnemius muscle. A notable reduction in mRNA expression levels of PINK1, PARKIN, citrate synthase, and cytochrome C oxidase genes was observed in the gastrocnemius muscle of the reserpine-treated group compared to the control group (*p* < 0.001). Furthermore, a significant decrease (*p* < 0.001) in FNDC gene expression was evident in the gastrocnemius muscle of mice in the reserpine-treated group relative to the control group (Table 3; Figs. 3, 4, 5, 6, and 7). In summary, reserpine injection led to a significant downregulation of mitochondrial functional markers, including PINK1, PARKIN, citrate synthase, and cytochrome C oxidase genes, as well as FNDC gene expression in the gastrocnemius muscle of mice in the reserpine-treated group compared to the control group (Table 3; Figs. 3, 2, 4, 5, 6, and 7).

**Table 3:** Relative expression of target genes compared to housekeeping gene in qPCR

FM/CoQ10	FM/EX	FM	EX/control	Control	Gene\group
16.3 ± 2.5^abc^	18.4 ± 1.8^bc^	3.0 ± 1.9^ab^	39.9 ± 6.0^a^	20.5 ± 1.9	Cytochrome C oxidase
32.3 ± 3.0^abcd^	47.2 ± 3.5^abc^	1.7 ± 0.9^ab^	82.9 ± 5.3^a^	26.4 ± 2.8	Citrate synthase
12.6 ± 1.3^bc^	11.0 ± 1.4^bc^	1.6 ± 0.8^ab^	22.1 ± 2.2^a^	12.2 ± 2.0	PINK
12.5 ± 1.1^abcd^	10.9 ± 1.2^abc^	3.7 ± 0.9^ab^	18.1 ± 1.1	17 ± 1.5	PARKIN
1.9 ± 1.0^bcd^	6.6 ± 1.2^abc^	0.6 ± 0.3^ab^	11.4 ± 1.4^a^	2.8 ± 0.8	Mitofusion 2
1.1 ± 0.5^abcd^	11.2 ± 1.3^abc^	0.9 ± 0.3^ab^	16.1 ± 2.9^a^	7.6 ± 1.2	FNDC5

The data are presented as mean ± SD. Statistical analysis was conducted using one-way ANOVA followed by Bonferroni post hoc testing

a: *p* value between control and other groups. b: *p* value between EX/control and (FM, FM/EX and FM/CoQ10). c: *p* value between FM and (FM/EX andFM/CoQ10). d: *p* value between FM/EX and FM/Co. Means with different superscripts indicate significant differences when *p* < 0.05

### Effect of resistance exercise on mRNA expression of mitochondrial markers and FNDC gene in the gastrocnemius muscle

After 4 weeks of climbing exercise, significant improvements were observed in the expression of mitochondrial markers and FNDC5 genes in the gastrocnemius muscle of C57Black/6 mice in the exercise-reserpine group. Specifically, a noteworthy increase in PINK mRNA expression was noted in exercise-reserpine group compared to the reserpine-treated (*p* < 0.001). However, this difference was not statistically significant when compared to the control mice (*p* = 0.476).

Regarding PARKIN, mitofusion2, and citrate synthase, the climbing exercise program resulted in higher mean mRNA expression levels of these genes in the exercise-reserpine group compared to the control and reserpine-treated groups (*p* < 0.001). Similarly, the mRNA expression of cytochrome C oxidase indicated a significant increase in mean mRNA expression levels in the exercise-reserpine group compared to the reserpine-treated group (*p* < 0.001), with no significant difference compared to the control group (*p* = 0.604). Furthermore, the mRNA expression of the FNDC5 gene exhibited a significant elevation in exercise-reserpine group C57Black/6 mice compared to the reserpine-treated and control groups (*p* < 0.001). To sum up, the 4-week climbing exercise regimen elicited notable enhancements in mitochondrial markers and FNDC5 gene expression within the gastrocnemius muscle of C57Black/6 mice. These findings underscore the potential benefits of exercise interventions in mitigating reserpine-induced alterations in gene expression profiles.

### Effect of CoQ10 administration on mRNA expression of mitochondrial markers and FNDC genes in the gastrocnemius muscle

The effects of CoQ10 administration (10 mg/kg, once daily) for 4 weeks on mitochondrial marker gene expression and FNDC gene expression in the gastrocnemius muscle of C57BL/6 mice are summarized in Table 3. CoQ10 supplementation enhanced mitochondrial marker gene expression without impacting FNDC gene expression. Significantly higher mean mRNA expression levels of PINK and Mitofusion2 genes were observed in the CoQ10-treated group compared to the reserpine-treated group (*p* < 0.001), although these differences were not statistically significant compared to the control group (*p* = 0.986, 0.404, respectively). Moreover, the mean mRNA expression levels of PARKIN and cytochrome C oxidase genes in the CoQ10-treated group were significantly elevated compared to the reserpine-treated group (*p* < 0.001) but were significantly lower compared to the control group (*p* < 0.001, < 0.05, respectively). Additionally, there was a significant increase in the mean mRNA expression levels of the citrate synthase gene following CoQ10 administration compared to the control and reserpine-treated groups (*p* < 0.05).

These findings suggest that CoQ10 supplementation enhances mitochondrial marker gene expression in the gastrocnemius muscle of mice, potentially contributing to improved mitochondrial function and cellular energy metabolism.

### Partial restoration of gastrocnemius muscle structural integrity in reserpine-treated mice by resistance exercise and coenzyme Q10 administration

**Fig. 8:**
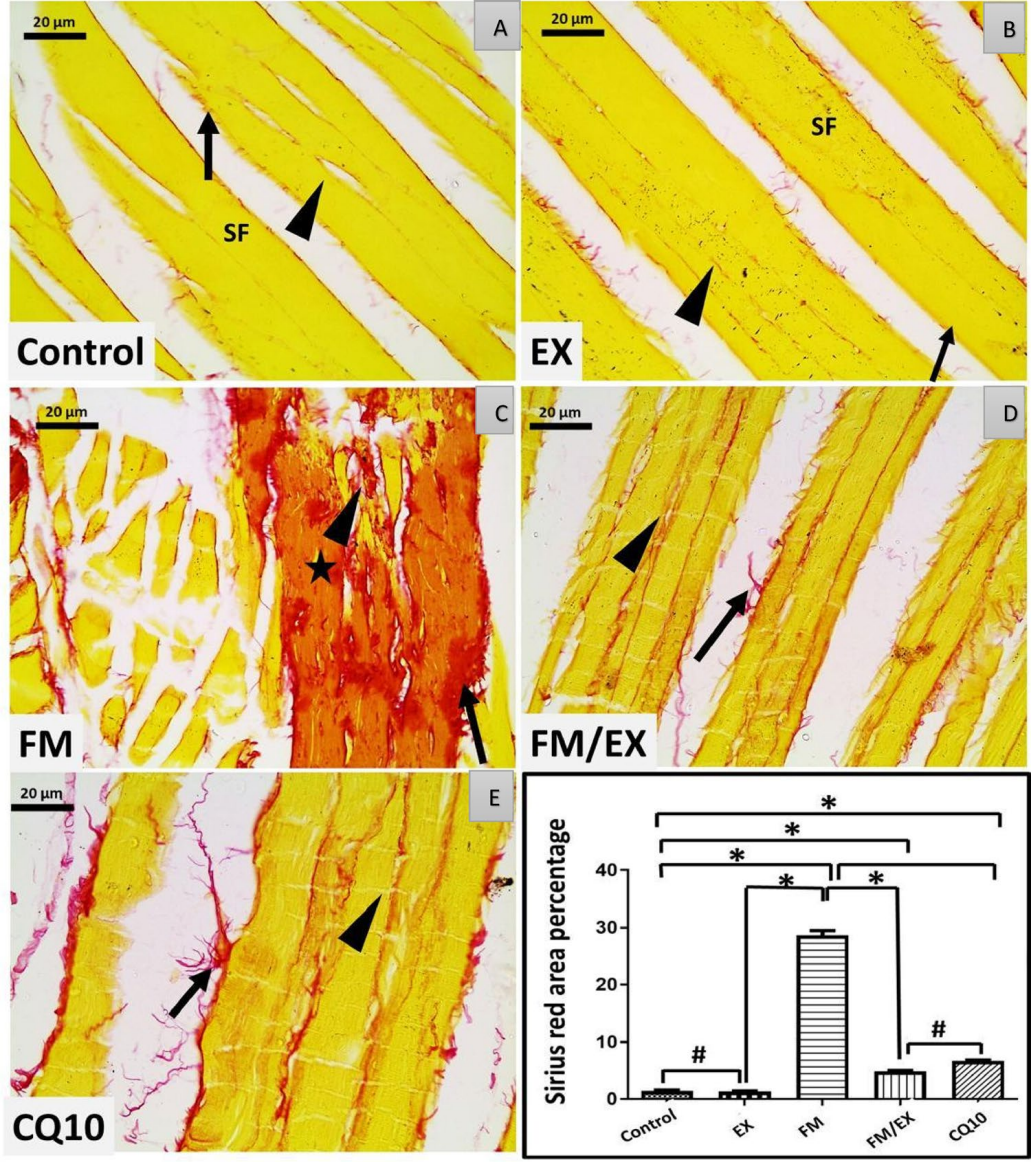
Histological assessment of grouped microscopic images for mice gastrocnemius muscle stained tissue sections stained with H&E from different groups. Control group: gastrocnemius skeletal muscle fibers (SF) of control group showing cylindrically arranged striated fibers (SF) with prominent striations (black star) in the eosinophilic sarcoplasm and multiple sub-sarcolemmal peripherally located flat vesicular nuclei. Group II: similar appearance to Group I with slightly increased muscle fiber diameter (double-headed arrow). Group III: showing destructed and widely separated muscle fibers (green star), loss of normal striation pattern (blue arrow), diluted congested blood vessels (BV), interstitial tissue edema (blue arrowhead), and inflammatory cellular infiltration (black arrowhead). Group IV: showing restoration of normal histological muscle fiber structure with parallel longitudinal skeletal muscle fibers (SF) and few connective tissue cells between fibers (yellow arrow). Group V: almost normal muscle fiber structure with parallel longitudinal skeletal muscle fibers (SF) and moderate connective tissue cells between fibers (yellow arrow)

Histological examination using H&E staining revealed that both resistance exercise and CoQ10 administration partially restored the structural integrity of the gastrocnemius skeletal muscle fibers (Fig. 8). In the control group, gastrocnemius muscle fibers appeared cylindrically arranged with prominent striations in the eosinophilic sarcoplasm and multiple sub-sarcolemmal peripherally located flat vesicular nuclei. Similarly, following the climbing exercise program in the exercise-control group, muscle fibers exhibited characteristics similar to those of the control group but with slightly increased diameter. However, reserpine-treated mice displayed disrupted and widely separated muscle fibers with a loss of normal striation patterns, diluted congested blood vessels, interstitial tissue edema, and inflammatory cellular infiltration. Notably, the climbing exercise program in mice injected with reserpine resulted in muscle fiber structures that closely resembled normal patterns. Furthermore, CoQ10 administration restored muscle fiber structure, with longitudinally oriented skeletal muscle fibers and fewer inflammatory connective tissue cells present between them. These findings highlight the potential of resistance exercise and CoQ10 supplementation in ameliorating reserpine-induced disruptions in gastrocnemius muscle morphology.

### Effects of reserpine injection, resistance exercise, and coenzyme Q10 administration on skeletal muscle collagen fibers’ area percentage

Assessment of gastrocnemius muscle interstitial collagen fibers’ formation and deposition stained with Sirius red revealed a marked increase in collagen fibers’ area percentage following reserpine injection, resistance exercise, and CoQ10 administration. Reserpine injection induced a significant increase in the mean area percentage of collagen muscle fibers compared to the control group (*p* < 0.001) (Fig. 8B). Conversely, resistance exercise resulted in a significant reduction in the mean area of collagen muscle fibers (4.8 ± 0.6976), exhibiting a statistically significant decrease compared to both reserpine-treated group and reserpine-exercise group (*p* = 0.000 and 0.027, respectively) (Fig. 8B). Similarly, CoQ10 administration led to a significant decrease in the mean area of collagen muscle fibers (6.67 ± 0.6734), with a statistically significant reduction observed compared to reserpine-treated group (*p* = 0) (Fig. 8B). Additionally, in reserpine-treated group, the mean area of collagen muscle fibers showed a significant decrease (0.845 ± 0.3382) compared to control group (*p* = 0.027) (Fig. 8B). To sum, reserpine injection resulted in a significant increase in collagen fibers formation and deposition within the gastrocnemius, whereas both resistance exercise and CoQ10 administration led to significant decreases, with particularly notable reductions observed in reserpine-treated groups compared to controls.

### Ultrastructural analysis of gastrocnemius muscle following reserpine injection and intervention

**Fig. 9:**
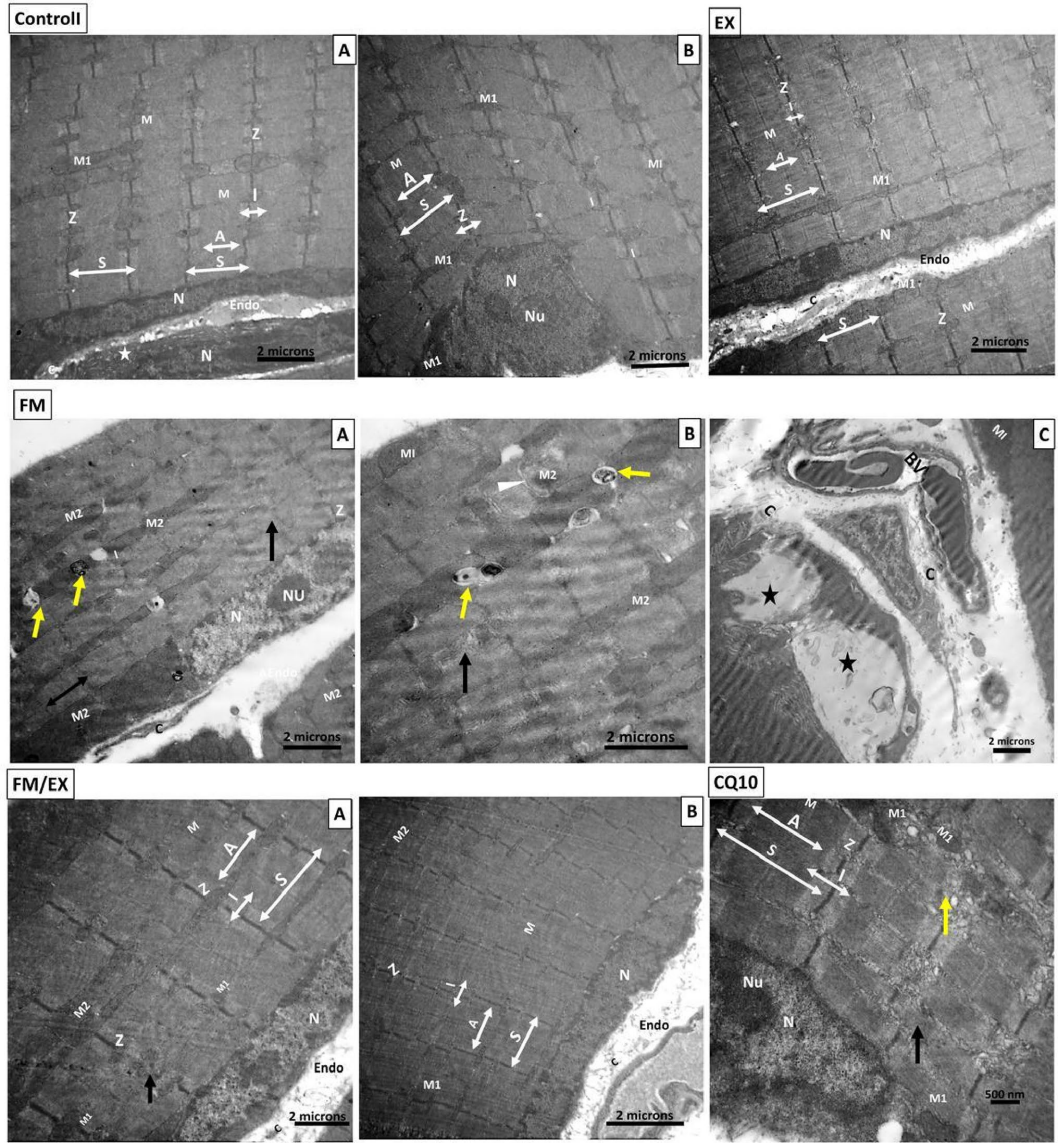
Grouped photomicrographs of longitudinal sections of mouse gastrocnemius muscle tissues sections stained with Sirius red, illustrating varying degrees of collagen fibers deposition. (GI) and (GII) depict scanty, red-stained collagen fibers in the endomysium between skeletal muscle fibers (black arrowhead) and in the perimysium surrounding the skeletal muscle fiber fascicle (black arrow). Marked collagen fiber deposition is evident in (GIII) replacing the within damaged fibers (black star). Conversely, in the exercise-treated fibromyalgia group (GIV) and coenzyme Q10-treated fibromyalgia group (GV), mild collagen fibrosis is observed in the endomysium (black arrowhead) and perimysium (black arrow) connective tissue (magnification ×400; scale bar: 20 µm)

Grouped photomicrographs of longitudinal sections of mouse gastrocnemius muscle tissues sections stained with Sirius red, illustrating varying degrees of collagen fibers deposition. GI and GII exhibited the typical ultrastructure of the gastrocnemius muscle, characterized by depict scanty, red-stained collagen fibers in the endomysium between skeletal muscle fibers (black arrowhead) and in the perimysium surrounding the skeletal muscle fiber fascicle (black arrow) (Fig. 9A, B).

Marked collagen fiber deposition is evident in (GIII) replacing the damaged fibers (black star) (Fig. 9C).

Conversely, in the exercise-treated fibromyalgia group (GIV) and coenzyme Q10-treated fibromyalgia group (GV) with mild collagen fibrosis is observed in the endomysium (black arrowhead) and perimysium (black arrow) connective tissue (magnification 400×; scale bar: 20 µm) (Fig. 9D, E).

**Fig. 10:**
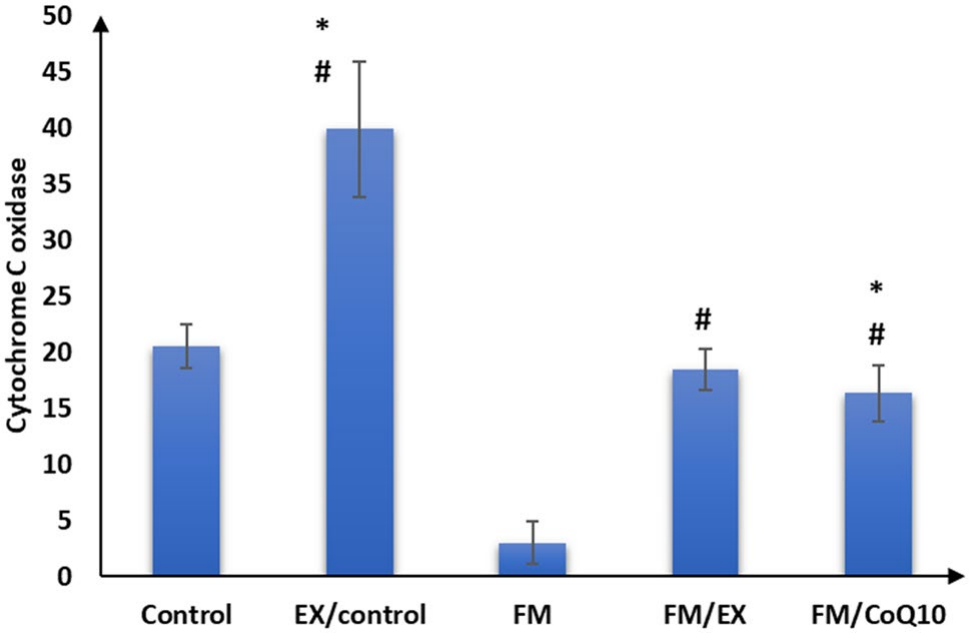
Electron microscopic ultrathin micrographs of longitudinal cut sections of gastrocnemius muscle. Gastrocnemius muscle of (GI) Group I (**A** and **B**): showing longitudinal ultrathin section revealing peripherally located sub-sarcolemma vesicular oval nucleus (N) with prominent nucleolus (Nu) and minimal collagen fibers (c) in the endomysium. Sarcomeres (S) with dark (A) and light (I) bands, Z lines (Z), and M lines are observed. Multiple sub-sarcolemma and interfibrillar mitochondria (M1) are evident. Gastrocnemius muscle of (GII) Group II: showing longitudinal ultrathin section displaying features like GI with peripherally located sub-sarcolemma vesicular oval nucleus (N), sarcomeres (S), Z lines (Z), and M lines. Multiple sub-sarcolemma and interfibrillar mitochondria (M1) are evident. C. Gastrocnemius muscle of FM group (**A**, **B** and **C**): showing longitudinal ultrathin section demonstrating peripherally located sub-sarcolemma vesicular oval nucleus (N) with prominent nucleolus (Nu). Muscle fibers are separated by wider endomysium space containing collagen fibers (c) and congested blood vessels (BV). Disturbed pattern of sarcomeres and interfibrillar vacuolation is observed. Gastrocnemius muscle of FM/EX Group (**A** and **B**): showing longitudinal section exhibiting nearly normal muscle fibers with normal banding pattern and normal mitochondria (M1). Minimal collagen fibers (endomysium) and satellite cells (S) are observed. Gastrocnemius muscle of CQ10 Group: showing longitudinal section displaying mostly regular muscle fibers with normal banding pattern. Some areas of disturbed pattern are observed, along with multiple sub-sarcolemma and interfibrillar mitochondria (M1)

Transmission electron microscope (TEM) examination unveiled distinctive ultra-structural features in the gastrocnemius muscle across the experimental groups. The control group exhibited the typical ultrastructure of the gastrocnemius muscle, characterized by well-defined sarcomeres with distinct dark and light bands, centrally located Z lines within the I band, and M lines positioned in the middle of the A band. Minimal connective tissue fibers were observed, and mitochondria displayed a typical arrangement with sub-sarcolemma and interfibrillar distribution, exhibiting intact outer membranes and discernible cristae (Fig. 10A, B).

In contrast, control mice subjected to the climbing exercise program displayed an ultrastructure like that of control group, with minimal collagen fibers observed between muscle fibers and numerous sub-sarcolemmal and interfibrillar mitochondria (M1) (Fig. 10EX). In reserpine-treated group, mice exhibited structurally altered and damaged muscle tissue, characterized by widened spaces between muscle fibers containing collagen fibers, congested blood vessels, and branched connective tissue cells. Sarcomeres displayed disrupted patterns, disorganized myofibrils, and numerous vacuolations with heterogeneous contents resembling autophagosomes and autolysosomes. Mega-mitochondria (M2) were observed surrounded by double-membrane autophagosomes and large vacuoles (Fig. 10FM A,B,C). Conversely, reserpine-exercise mice demonstrated nearly normal muscle fibers with a typical banding pattern and mitochondria, albeit with small areas of disrupted patterns and a few mega-mitochondria. Minimal connective tissue fibers (endomysium) and collagen fibers were observed, along with satellite cells outside the sarcolemma (Fig. 10FM/EX A,B). There was a statistically significant increase in the number of abnormal mitochondria in the gastrocnemius muscles of mice in the reserpine-treated group compared to the control group (*p* < 0.0001). The number of abnormal mitochondria decreased significantly in both reserpine-exercise group (*p* < 0.0001) and CoQ10-treated mice (*p* < 0.0001). Finally, in CoQ10-treated mice displayed a mostly regular pattern of muscle fibers with normal banding, along with areas of disrupted patterns and numerous sub-sarcolemma and interfibrillar mitochondria (M1).

Taken together, TEM examination of the gastrocnemius muscle ultrastructure revealed distinct differences among the experimental groups: The control group and the exercise-control group exhibited typical muscle architecture, whereas the reserpine-treated group displayed significant ultra-structural muscle fiber damage and fibrosis, the reserpine-exercise group showed partial recovery, and CoQ10 group displayed mixed patterns of restoration and disruption.

## Discussion

The study demonstrates the significant impact of reserpine on inducing behavioral and pathophysiological changes akin to fibromyalgia. Following reserpine injection, mice exhibited heightened pain sensitivity, reduced locomotion, increased anxiety-like behavior, depression-like symptoms, and decreased muscle strength. Additionally, the observed decrease in muscle strength underscores reserpine’s debilitating effects on skeletal muscle function. Notably, the study highlights the efficacy of resistance exercise and CoQ10 supplementation in mitigating reserpine-induced alterations, restoring pain sensitivity, locomotion, anxiety and depression-like behaviors, and muscle strength to baseline levels. Following these interventions, enhanced expression of mitochondrial marker genes suggests improved mitochondrial function, including enhancements in cellular energy metabolism and mitophagy. Moreover, histological analysis reveals partial gastrocnemius muscle fiber integrity restoration, indicating potential regenerative effects in mitigating fibromyalgia-related muscle pathology. These findings underscore the multifaceted therapeutic effects of resistance exercise and CoQ10 supplementation on fibromyalgia pathology, warranting further research to optimize therapeutic protocols.

Fibromyalgia, a prevalent musculoskeletal pain condition, significantly impacts patients’ productivity and quality of life (López-Muñoz et al. 2023). Expanding upon previous research implicating reserpine in fibromyalgia-related alterations, our study demonstrated that three consecutive days of reserpine injection increased pain sensitivity and induced depression- and anxiety-like behaviors while decreasing locomotion, mirroring symptoms identified in fibromyalgia syndrome (FMS) (Yao et al. 2020) and animal models post-reserpine injection (Blasco-Serra et al. 2015; Nagakura et al. 2009). Consistent with prior reports linking reserpine to muscle weakness, muscle strength decreased after reserpine injection, as evidenced by weights and inverted screen tests, aligning with the previous findings (Tharwat et al. 2023). Although the exact etiology of fibromyalgia remains elusive, evidence suggests mitochondrial dysfunction may play a role in its pathophysiology (Siracusa et al. 2021). Mitochondria, dynamic organelles, undergo morphological changes regulated by fusion, fission, and mitophagy in response to metabolic and environmental cues (Muñoz et al. 2023). Our investigation into mitochondrial functions revealed decreased mRNA expression of cytochrome c oxidase and citrate synthase post-reserpine injection, essential enzymes in mitochondrial respiration (Dmitry 2021), shedding light on mitochondrial involvement in decreased muscle strength. These findings underscore the potential contribution of mitochondrial dysfunction to fibromyalgia pathology, deserving further exploration.

Furthermore, we observed a decrease in mitofusin2 mRNA expression in reserpine-treated mice. This finding is inconsistent with a previous study (Favero et al. 2019), which reported weak expression of Mfn2 in a reserpine-treated model. Mitofusin-2 (Mfn2) is a multifunctional protein known to regulate mitochondrial fusion–fission processes and several cellular functions, including mitochondrial metabolism, biogenesis, signaling, and apoptosis, by maintaining ER–mitochondria contact sites (Kulkarni et al. 2023). In cases of mitochondrial dysfunction, mitochondrial fission and mitophagy play crucial roles in regaining metabolic homeostasis and preserving mitochondrial integrity (He et al. 2020). We also observed decreased mRNA expression of mitochondrial mitophagy markers (PINK1 and Parkin) in reserpine-treated mice compared to controls. This suggests that mitochondrial damage induced by FM may surpass the capacity for selective cleavage and clearance. PINK1 and Parkin are proteins in the mitophagy-mediated pathway that regulate mitochondrial function (Han et al. 2020). This finding aligns with a previous study (Han et al. 2020). Additionally, our study’s abnormal mega-mitochondria accumulated in disturbed sarcomeres observed under electron transmission microscopy further supports this perspective (Liang et al. 2023).

Moreover, we noted a decrease in mitofusin2 mRNA expression in reserpine-treated mice, which contradicts prior findings (Favero et al. 2019) reporting weak Mfn2 expression in reserpine-treated models. Mitofusin-2 (Mfn2) plays multifunctional roles, regulating mitochondrial fusion–fission dynamics and influencing cellular processes such as mitochondrial metabolism, biogenesis, signaling, and apoptosis via the maintenance of ER-mitochondria contact sites. In instances of mitochondrial dysfunction, mitochondrial fission, and mitophagy become crucial for restoring metabolic homeostasis and preserving mitochondrial integrity. Our study revealed decreased mRNA expression of mitochondrial mitophagy markers (PINK1 and Parkin) in Fibromyalgia compared to control mice, suggesting that FM-induced mitochondrial damage may surpass the capacity for selective cleavage and clearance. PINK1 and PARKIN participate in mitophagy-mediated pathways regulating mitochondrial function, corroborating previous findings. Additionally, the presence of abnormal mega-mitochondria within disrupted sarcomeres, as observed via electron transmission microscopy in our study, further supports this notion (Favero et al. 2024). These results underscore the intricate interplay between mitochondrial dynamics and fibromyalgia pathology, warranting deeper investigation.

Furthermore, our results revealed a decrease in mRNA expression of the FNDC5 gene in the gastrocnemius muscle of the reserpine-treated group. Previous studies have reported a positive correlation between serum irisin levels and muscle strength and physical performance in postmenopausal women (Liang et al. 2023). Additionally, Sarangi et al. (2024) observed that postmenopausal women with lower serum irisin levels have weaker muscle strength. Ge et al. (2023) reported that FNDC5 deficiency disrupts mitochondrial dynamics and function, while others demonstrated mitochondrial dysfunction along with reduced mRNA expression of FNDC5 in muscle biopsies of diabetic and idiopathic inflammatory myopathies.

Exercise training is an economical and efficient nonpharmacological intervention targeting mitochondria, inducing beneficial mitochondrial adaptations, and increasing mitochondrial quality and content (He et al. 2020). In line with this, we hypothesized that exercise could improve mitochondrial health and alleviate reserpine-induced FM in black mice. We subjected reserpine-induced FM mice to a resistance climbing exercise program for 4 weeks to test this hypothesis. Then, we evaluated their behavioral performance and muscle strength at the end of the 4-week program. We observed improvements in pain sensitivity, locomotion, anxiety, and depression-like behaviors, consistent with the previous findings (Dixit et al. 2023; Jung et al. 2023). Interestingly, muscle strength showed improvement compared to measurements of the reserpine-treated group. Additionally, Jang et al. reported that resistance exercise for 14 days completely blocked the loss of muscle strength induced by dexamethasone and increased muscle strength beyond that of the control group (Jang et al. 2022).

PINK1 and PARKIN are involved in mitophagy, a process by which damaged mitochondria are targeted for degradation (Ge et al. 2020). Interestingly, the climbing exercise program contributed to the elevation of Cytochrome C oxidase, citrate synthase, PINK1, Parkin, and mitofusin2 expression, as well as to the histopathological improvement of sarcomeres, collagen fibers, and nearly normal mitochondria in the gastrocnemius muscle of the current study. These results are consistent with others (Romero et al. 2019; Long et al. 2022), suggesting that the beneficial role of exercise in mitochondriopathy evidenced in FM-induced mice may be through repairing mitochondrial functions and mitophagy. Moreover, the increased expression of FNDC5 in the exercised group in this study further supports the involvement of FNDC5 in the influence of exercise on mitochondrial adaptations in FM. FNDC5/irisin, mainly expressed and secreted by skeletal muscle (Leger et al. 2024), is primarily known for its role in producing irisin, a hormone involved in energy metabolism and exercise-induced adaptations. Exercise can influence its expression and impact metabolic processes (Wrann 2015). FNDC5/irisin has been shown to protect mitochondrial function in cardiomyocytes against ischemia/reperfusion injury (Grzeszczuk et al. 2024). Similarly, Li et al. (2021) reported that resistance exercise can improve cardiac function by activating the irisin/FNDC5-PINK1/Parkin-LC3/P62 pathway, regulating mitophagy.

In addition, previous studies have suggested that mitochondrial dysfunctions found in FM could result from a deficiency of coenzyme Q10 or ubiquinone-10 involved in the mitochondrial respiratory chain (Cordaro et al. 2022). In this study, we investigated the effect of CoQ10 administration (10 mg/kg, once daily) for 4 weeks. We observed significant improvements in pain sensitivity, locomotion, anxiety, and depression-like behaviors. Furthermore, increased muscle strength was also observed in the current study, which is consistent with the previous findings (Campisi and Motta 2022). Additionally, after CoQ10 administration, there was an elevation in mRNA expression of cytochrome C oxidase, citrate synthase, PINK, PARKIN, and mitofusin 2 in the gastrocnemius muscle of mice. Wang et al. reported that CoQ10 may protect the functions of the mitochondria complex by up-regulating the marker of mitochondria enrichment, the activity of citrate synthase in skeletal muscles (Wang et al. 2019). Moreover, Zhang et al. (2021) reported that CoQ10 treatment promoted mitophagy through increased expression of mitophagy proteins (PINK1 and PARKIN). The increased expression of FNDC5 in this study can explain the improvement of parameters after Exercise. The close relationship of FNDC5/irisin with mitochondrial genes and proteins that regulate mitochondrial function has been reported (Zhang et al. 2024).

On the other hand, FNDC expression was not affected by CoQ10 administration. Favero et al. (2019) reported that the ultra-structural evaluation of reserpine-induced myalgia (RIM) gastrocnemius muscles showed different sarcomeric lengths, heterogeneous and altered interfibrillar and sub-sarcolemmal mitochondria, and dilated and deformed sarcoplasmic reticulum that appeared associated with mitochondria. Control muscle cells showed normal nuclei with active nucleoli, regular mitochondria distribution, and sarcoplasmic reticulum. Rats treated with reserpine had a weak but insignificant recovery of the gastrocnemius ultrastructure. Also, it was found that the diameter of gastrocnemius myotubes decreased significantly in the experimental group treated with reserpine compared with controls (ANOVA, *p* ≤ 0.05) (Grzeszczuk et al. 2024). Using electron microscopy, Kitaoka et al. examined the morphological alterations of mitochondria in skeletal muscle. They found that physical exercise could alleviate an increased appearance of damaged mitochondria with disrupted cristae structure in cancer cachexia mice (Kitaoka et al. 2021).

Our study had some limitations; we could not investigate more targets (e.g., monoamine levels). Additionally, further investigations are required to evaluate the effect of exercise on mitochondrial fission in FM-induced models. Resistance exercise and CoQ10 appear to play an essential role for FM patients. PINK/PARKIN pathway activation appears to be an important mechanism for improving FM (Zhao et al. 2020). Drugs activating this pathway would be of great value as a treatment for FM (Silvian 2022).

## Conclusion

This study investigates therapeutic strategies for fibromyalgia using a reserpine-induced mice model. The study demonstrates that both resistance exercise and CoQ10 supplementation significantly alleviate fibromyalgia-like symptoms, with resistance exercise conferring a markedly superior benefit. The enhanced efficacy of exercise is likely mediated through the restoration of mitochondrial function via activation of the PINK1–PARKIN mitophagy pathway, a process potentiated by the myokine IRISIN. Our findings posit the targeting of mitochondrial dynamics, specifically through exercise-induced mitophagy, as a promising therapeutic strategy for fibromyalgia management.

## Supplementary Information

Below is the link to the electronic supplementary material.

Supplementary file1 (DOCX 67 kb)

## Data Availability

Data are available upon reasonable request from corresponding author.

## Abbreviations

FM: Fibromyalgia
CoQ10: Coenzyme Q10
PINK1: PTEN-induced kinase 1
FNDC5: Fibronectin type III domain-containing protein 5
Mfn: Mitofusins
p62: Protein sequestosome1
LC3: Light chain 3
CS: Citrate synthase
HPT: Hot-plate test
FST: Forced swim test
OFT: Open-field test
PVC: Polyvinyl chloride
qPCR: Quantitative real-time polymerase chain reaction
FNDC5: Fibronectin type III domain-containing protein 5
M1: Interfibrillar mitochondria
M2: Mega-mitochondria
Mfn2: Mitofusin-2
ANOVA: Analysis of variance
