# Supplementary material for: Involvement of the PINK1/PARKIN pathway in enhancing mitochondrial function and mitophagy in reserpine-induced fibromyalgia mice through strength exercise and coenzyme Q10
Source: Eur J Appl Physiol. 2025 Dec 2;126(4):2219–34. doi: 10.1007/s00421-025-05990-0 (PMC13171688; doi:10.1007/s00421-025-05990-0)
Supplement: Supplementary file 1 — Supplementary file1 (DOCX 67 kb) [file 421_2025_5990_MOESM1_ESM.docx]

**Figure 11; the mean of abnormal mitochondria in the control, control\exercise, reserpine-treated \exercise, CoQ10 groups.**


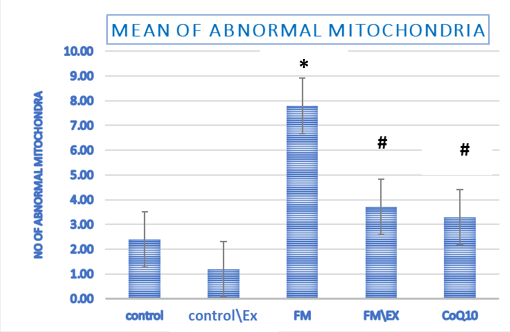


* Means significant compared to control group, # means significant compared to FM group, p < 0.05.
